# Supplementary material for: A network correspondence toolbox for quantitative evaluation of novel neuroimaging results
Source: Nat Commun. 2025 Mar 25;16:2930. doi: 10.1038/s41467-025-58176-9 (PMC11937327; doi:10.1038/s41467-025-58176-9)

Supplementary Information: A network correspondence toolbox for quantitative evaluation of novel neuroimaging results

Supplementary Table 1. Sixteen widely used brain atlases in fsaverage6 surface space, fs_LR_32k surface space, and MNI space were included in the NCT. The first column shows the studies from which the atlases were obtained. The second column shows the specific atlas we included in the NCT.

Abbreviation	Reference	Atlas
Laird2011-20	Laird2011	Laird2011 20-node ICA-BrainMap maps
Yeo2011-7	Yeo2011	Yeo2011 7 networks
Yeo2011-17	Yeo2011	Yeo2011 17 networks
Shirer2012-90-14	Shirer2012	Shirer2012 90-ROI 14 networks
Smith2013-25	Smith2013	HCP 25-node ICA maps
Shen2013-268-8	Shen2013	Shen2013 268-ROI with 8 networks
Shen2013-368-8	Shen2013	Shen2013 368-ROI with 8 networks
Laumann2015-12	Laumann2015	Laumann2015 12 networks (Power2011)
Miller2016-25	Miller2016	UK Biobank 25-node ICA maps
Gordon2016-286-12	Gordon2016	Gordon2016 286-ROI with 12 networks
Gordon2017-17	Gordon2017	Gordon2017 17 networks
Schaefer2018-200+Kong2021-17	Schaefer2018	Schaefer2018 200-ROI with Kong2021 17 networks
Schaefer2018-200+Yeo2011-17	Schaefer2018	Schaefer2018 200-ROI with Yeo2011 17 networks
Schaefer2018-400+Kong2021-17	Schaefer2018	Schaefer2018 400-ROI with Kong2021 17 networks
Schaefer2018-400+Yeo2011-17	Schaefer2018	Schaefer2018 400-ROI with Yeo2011 17 networks
Glasser2016-360+Ji2019-12	Ji2019	Glasser2016 360-ROI with Ji2019 12 Cole-Anticevic networks

Supplementary Table 2. Network-to-network spatial correspondence between all 136 network pairs from the Yeo2011-17 (rows) and Gordon2017-17 (columns) atlases (Dice coefficients and corresponding *p*-values).

	**Default**	**LatVis**	**FrontPar**	**MedVis**	**DorsAttn**	**Premotor**	**Language**	**Salience**	**CingOperc**
**VisualA**	0.0000 (p=0.9461)	0.7311 (p=0.0010)	0.0000 (p=0.9131)	0.0858 (p=0.1548)	0.0682 (p=0.3437)	0.0000 (p=0.5225)	0.0000 (p=0.6763)	0.0000 (p=0.4665)	0.0000 (p=0.9251)
**VisualB**	0.0005 (p=0.9191)	0.3164 (p=0.0799)	0.0000 (p=0.9091)	0.6462 (p=0.0030)	0.0004 (p=0.6993)	0.0000 (p=0.5145)	0.0000 (p=0.6593)	0.0000 (p=0.4515)	0.0000 (p=0.9081)
**SomatomotorA**	0.0000 (p=0.9860)	0.0000 (p=0.6523)	0.0000 (p=0.9790)	0.0000 (p=0.3566)	0.0000 (p=0.8821)	0.0738 (p=0.2318)	0.0000 (p=0.8272)	0.0000 (p=0.5694)	0.0207 (p=0.7512)
**SomatomotorB**	0.0000 (p=0.9860)	0.0000 (p=0.5814)	0.0000 (p=0.9680)	0.0000 (p=0.3536)	0.0000 (p=0.8122)	0.0108 (p=0.4076)	0.0098 (p=0.6384)	0.0000 (p=0.5415)	0.0759 (p=0.4605)
**DorsAttnA**	0.0011 (p=0.9860)	0.1205 (p=0.2198)	0.0307 (p=0.6444)	0.0000 (p=0.4336)	0.6204 (p=0.0010)	0.0098 (p=0.4965)	0.0222 (p=0.5534)	0.0000 (p=0.6434)	0.0011 (p=0.9580)
**DorsAttnB**	0.0000 (p=1.0000)	0.0000 (p=0.6973)	0.0059 (p=0.9321)	0.0000 (p=0.4595)	0.2730 (p=0.0010)	0.6132 (p=0.0010)	0.0101 (p=0.7303)	0.0000 (p=0.6364)	0.0736 (p=0.4715)
**Sal/VenAttnA**	0.0004 (p=1.0000)	0.0000 (p=0.8731)	0.0031 (p=0.9750)	0.0000 (p=0.6054)	0.0018 (p=0.9590)	0.0114 (p=0.6543)	0.0099 (p=0.8312)	0.0120 (p=0.6134)	0.7310 (p=0.0010)
**Sal/VenAttnB**	0.0022 (p=0.9970)	0.0000 (p=0.8172)	0.1058 (p=0.2078)	0.0000 (p=0.5724)	0.0063 (p=0.8631)	0.0000 (p=0.7912)	0.0556 (p=0.3676)	0.3719 (p=0.0020)	0.2913 (p=0.0010)
**LimbicA**	0.0162 (p=0.6653)	0.0029 (p=0.3237)	0.0354 (p=0.4066)	0.0000 (p=0.2038)	0.0009 (p=0.5734)	0.0000 (p=0.4266)	0.0060 (p=0.4915)	0.0000 (p=0.3636)	0.0127 (p=0.5634)
**LimbicB**	0.0879 (p=0.2078)	0.0002 (p=0.3676)	0.0757 (p=0.2008)	0.0000 (p=0.2068)	0.0000 (p=0.5914)	0.0000 (p=0.4106)	0.0000 (p=0.5075)	0.0004 (p=0.3247)	0.0095 (p=0.5205)
**ControlC**	0.0796 (p=0.1658)	0.0000 (p=0.4815)	0.0036 (p=0.7133)	0.0000 (p=0.2517)	0.0229 (p=0.4476)	0.0000 (p=0.5135)	0.0000 (p=0.6793)	0.0000 (p=0.4256)	0.0296 (p=0.4835)
**ControlA**	0.0026 (p=0.9960)	0.0000 (p=0.9271)	0.5897 (p=0.0010)	0.0000 (p=0.6424)	0.1430 (p=0.0889)	0.0000 (p=0.8901)	0.0179 (p=0.7622)	0.0000 (p=0.8222)	0.0066 (p=0.9770)
**ControlB**	0.2171 (p=0.0050)	0.0000 (p=0.8981)	0.3880 (p=0.0010)	0.0000 (p=0.5824)	0.0052 (p=0.9041)	0.0000 (p=0.8751)	0.0202 (p=0.6993)	0.0497 (p=0.2697)	0.0029 (p=0.9900)
**TempPar**	0.0061 (p=0.8521)	0.0000 (p=0.6474)	0.0000 (p=0.9381)	0.0000 (p=0.3716)	0.0000 (p=0.8182)	0.0000 (p=0.5754)	0.6375 (p=0.0010)	0.0000 (p=0.5385)	0.0091 (p=0.6513)
**DefaultC**	0.0518 (p=0.3267)	0.0024 (p=0.4765)	0.0013 (p=0.8651)	0.0255 (p=0.2178)	0.0003 (p=0.7902)	0.0000 (p=0.5774)	0.0090 (p=0.5235)	0.0000 (p=0.5055)	0.0004 (p=0.9381)
**DefaultA**	0.5622 (p=0.0010)	0.0000 (p=0.9161)	0.0044 (p=0.9660)	0.0000 (p=0.5984)	0.0000 (p=0.9830)	0.0000 (p=0.9181)	0.0149 (p=0.7682)	0.0786 (p=0.1648)	0.0000 (p=0.9990)
**DefaultB**	0.3694 (p=0.0010)	0.0000 (p=0.8641)	0.0193 (p=0.8901)	0.0000 (p=0.6004)	0.0003 (p=0.9680)	0.0000 (p=0.8392)	0.3259 (p=0.0020)	0.0831 (p=0.1449)	0.0032 (p=0.9830)

Supplementary Table 2 (continued).

	HandSM	FaceSM	Auditory	AntMTL	PostMTL	ParMemory	Context	FootSM
**VisualA**	0.0000 (p=0.5994)	0.0000 (p=0.3287)	0.0000 (p=0.4116)	0.0000 (p=0.2637)	0.0000 (p=0.2408)	0.0000 (p=0.2777)	0.0043 (p=0.4565)	0.0000 (p=0.3217)
**VisualB**	0.0000 (p=0.5944)	0.0000 (p=0.3137)	0.0000 (p=0.4196)	0.0000 (p=0.2517)	0.0030 (p=0.1718)	0.0310 (p=0.1898)	0.0635 (p=0.2058)	0.0000 (p=0.3277)
**SomatomotorA**	0.5121 (p=0.0010)	0.0332 (p=0.2258)	0.0161 (p=0.3197)	0.0000 (p=0.4056)	0.0000 (p=0.3127)	0.0000 (p=0.3636)	0.0000 (p=0.7043)	0.6892 (p=0.0010)
**SomatomotorB**	0.0076 (p=0.4875)	0.4738 (p=0.0020)	0.6948 (p=0.0010)	0.0000 (p=0.3487)	0.0000 (p=0.2987)	0.0000 (p=0.3516)	0.0000 (p=0.6883)	0.0000 (p=0.3716)
**DorsAttnA**	0.0016 (p=0.7223)	0.0000 (p=0.4885)	0.0000 (p=0.6454)	0.0000 (p=0.4665)	0.0000 (p=0.3596)	0.0186 (p=0.2787)	0.0496 (p=0.2617)	0.0000 (p=0.5195)
**DorsAttnB**	0.0543 (p=0.2697)	0.0102 (p=0.3956)	0.0000 (p=0.6783)	0.0000 (p=0.4815)	0.0000 (p=0.3916)	0.0000 (p=0.4246)	0.0000 (p=0.7652)	0.0012 (p=0.5175)
**Sal/VenAttnA**	0.0114 (p=0.6194)	0.0094 (p=0.4705)	0.0213 (p=0.4855)	0.0023 (p=0.5734)	0.0000 (p=0.5175)	0.0027 (p=0.5185)	0.0000 (p=0.8801)	0.0100 (p=0.5085)
**Sal/VenAttnB**	0.0000 (p=0.8781)	0.0000 (p=0.5774)	0.0000 (p=0.7223)	0.0000 (p=0.6014)	0.0000 (p=0.4735)	0.0063 (p=0.4346)	0.0000 (p=0.8482)	0.0000 (p=0.6394)
**LimbicA**	0.0237 (p=0.2498)	0.0000 (p=0.2148)	0.0000 (p=0.3457)	0.7469 (p=0.0010)	0.0183 (p=0.1159)	0.0000 (p=0.2148)	0.0000 (p=0.4695)	0.0000 (p=0.2488)
**LimbicB**	0.0000 (p=0.4446)	0.0000 (p=0.2338)	0.0000 (p=0.3087)	0.0000 (p=0.1988)	0.0000 (p=0.1538)	0.0000 (p=0.1838)	0.0000 (p=0.4086)	0.0000 (p=0.2398)
**ControlC**	0.0000 (p=0.5325)	0.0000 (p=0.3237)	0.0000 (p=0.4096)	0.0000 (p=0.2727)	0.0000 (p=0.1858)	0.4955 (p=0.0010)	0.0305 (p=0.2597)	0.0000 (p=0.3397)
**ControlA**	0.0000 (p=0.9201)	0.0003 (p=0.6484)	0.0000 (p=0.8072)	0.0000 (p=0.6703)	0.0000 (p=0.5375)	0.0000 (p=0.6114)	0.0031 (p=0.7632)	0.0000 (p=0.7502)
**ControlB**	0.0000 (p=0.8851)	0.0000 (p=0.6224)	0.0000 (p=0.7652)	0.0000 (p=0.6553)	0.0000 (p=0.5205)	0.0000 (p=0.5784)	0.0069 (p=0.6903)	0.0000 (p=0.7123)
**TempPar**	0.0025 (p=0.5305)	0.0000 (p=0.3576)	0.0358 (p=0.1748)	0.0000 (p=0.4176)	0.0000 (p=0.2747)	0.0000 (p=0.3307)	0.0000 (p=0.5844)	0.0000 (p=0.4595)
**DefaultC**	0.0000 (p=0.6384)	0.0000 (p=0.3776)	0.0000 (p=0.4725)	0.0253 (p=0.2108)	0.0661 (p=0.0340)	0.0000 (p=0.3327)	0.6406 (p=0.0010)	0.0000 (p=0.3996)
**DefaultA**	0.0000 (p=0.8981)	0.0000 (p=0.6783)	0.0000 (p=0.8062)	0.0000 (p=0.6643)	0.0000 (p=0.4975)	0.0543 (p=0.2068)	0.0425 (p=0.3227)	0.0000 (p=0.7612)
**DefaultB**	0.0000 (p=0.8911)	0.0000 (p=0.6294)	0.0000 (p=0.7672)	0.0309 (p=0.3896)	0.0000 (p=0.5235)	0.0038 (p=0.5095)	0.0000 (p=0.8721)	0.0000 (p=0.6843)

Supplementary Table 3. Network spatial correspondence between HCP working memory task contrast (2BK vs. 0BK) and networks from 8 atlases (Dice coefficient and corresponding *p*-values).

Glasser2016-360+Ji2019-12

Name	Dice	P value
Visual1	0.0031	0.7423
Visual2	0.0072	0.8911
Somatomotor	0.0001	0.997
CingOperc	0.225	0.0999
DorsAttn	0.2619	0.005*
Language	0.0455	0.6304
FrontPar	0.4751	0.001*
Auditory	0.0001	0.7253
Default	0.0561	0.9381
PostMulti	0.0251	0.4016
VentMulti	0.0001	0.6593
OrbitAffective	0.0006	0.5904
-	–	–
-	–	–
-	–	–
-	–	–
-	–	–

Schaefer2018-400+Kong2019-17

Name	Dice	P value
DefaultA	0.023	0.9161
DefaultB	0.0394	0.8102
DefaultC	0.1006	0.2308
Language	0.0583	0.4795
ControlA	0.3119	0.001*
ControlB	0.2559	0.001*
ControlC	0.1461	0.032*
Sal/VenAttnA	0.0388	0.7852
Sal/VenAttnB	0.1546	0.0979
DorsAttnA	0.221	0.011*
DorsAttnB	0.0406	0.6024
Auditory	0.0001	0.9111
SomatomotorA	0.0001	0.952
SomatomotorB	0.0003	0.9191
VisualA	0.0289	0.7782
VisualB	0.0032	0.8641
VisualC	0.0018	0.5604

Shirer2013-90-14

Name	Dice	P value
AntSal	0.1876	0.009*
Auditory	0.0001	0.7762
DorsalDMN	0.002	0.9091
HighVisual	0.0001	0.7183
Language	0.0049	0.7473
LECN	0.0874	0.042*
PostSal	0.0521	0.2348
Precuneus	0.078	0.1149
PrimVisual	0.0001	0.5664
RECN	0.2364	0.001*
Sensorimotor	0.0001	0.8352
VentralDMN	0.1819	0.021*
Visuospatial	0.1994	0.041*
-	–	–
-	–	–
-	–	–
-	–	–

Yeo2011-17

Name	Dice	P value
VisualA	0.0027	0.8262
VisualB	0.002	0.7942
SomatomotorA	0.0001	0.958
SomatomotorB	0.0001	0.9341
DorsAttnA	0.097	0.3277
DorsAttnB	0.1206	0.2507
Sal/VenAttnA	0.0261	0.9051
Sal/VenAttnB	0.2699	0.001*
LimbicA	0.0001	0.7972
LimbicB	0.0023	0.6633
ControlC	0.0986	0.1249
ControlA	0.34	0.001*
ControlB	0.3622	0.001*
TempPar	0.0067	0.7103
DefaultC	0.0077	0.7003
DefaultA	0.0534	0.7093
DefaultB	0.037	0.8102

Shen2013-268-8

Name	Dice	P value
MedFront	0.1151	0.5365
FrontPar	0.3676	0.007*
Default	0.0495	0.7383
Motor	0.0701	0.8981
VisualA	0.028	0.6783
VisualB	0.0055	0.5844
VisAssoc	0.1388	0.3337
SalSubcor	0.2523	0.025*
-	–	–
-	–	–
-	–	–
-	–	–
-	–	–
-	–	–
-	–	–
-	–	–
-	–	–

Laird2011-20

Name	Dice	P value
Emo/Interoception1	0.0139	0.9331
Emo/Interoception2	0.104	0.6004
Emo/Interoception3	0.0524	0.5594
Emo/Interoception4	0.1775	0.3876
Mot/Visspatial1	0.2556	0.1489
Mot/Visspatial2	0.4077	0.003*
Mot/Visspatial3	0.1743	0.4575
Mot/Visspatial4	0.0404	0.9131
Visual1	0.0794	0.8881
Visual2	0.0398	0.6793
Visual3	0.0508	0.7103
DivergentCog1	0.1651	0.6184
DivergentCog3	0.3949	0.005*
DivergentCog4	0.0423	0.8631
DivergentCog5	0.1287	0.5544
DivergentCog6	0.3031	0.001*
-	–	–

Supplementary Table 3 (continued).

Laumann2015-12			Gordon2017-17		
Name	Dice	P value	Name	Dice	P value
Default	0.094	0.6364	Default	0.0972	0.6953
Visual	0.2123	0.2228	LatVis	0.2355	0.1638
FrontPar	0.1293	0.2677	FrontPar	0.1321	0.3087
DorsAttn	0.1371	0.2697	MedVis	0.0024	0.6743
VentAttn	0.3729	0.002*	DorsAttn	0.1873	0.1299
Salience	0.0001	0.7253	Premotor	0.0004	0.9201
CingOperc	0.0344	0.8671	Language	0.3926	0.002*
SM	0.0001	0.981	Salience	0.0039	0.7732
LateralSM	0.0001	0.7922	CingOperc	0.0329	0.8781
Auditory	0.0004	0.8332	HandSM	0.0001	0.97
MedPar	0.0001	0.7223	FaceSM	0.0001	0.7552
ParOcc	0.1264	0.014*	Auditory	0.0007	0.8591
-	–	–	AntMTL	0.0329	0.3536
-	–	–	PostMTL	0.0007	0.5594
-	–	–	ParMemory	0.0001	0.7283
-	–	–	Context	0.1473	0.029*
-	–	–	FootSM	0.0001	0.8651

Supplementary Table 4. Network spatial correspondence between HCP social task contrast (theory of mind vs. random) and networks from 8 atlases (Dice coefficient and corresponding *p*-values).

Glasser2016-360+Ji2019-12			Schaefer2018-400+Kong2019-17			Shirer2013-90-14		
Name	Dice	P value	Name	Dice	P value	Name	Dice	P value
Visual1	0.0406	0.3886	DefaultA	0.0923	0.4256	AntSal	0.0085	0.7063
Visual2	0.2733	0.1499	DefaultB	0.1119	0.2787	Auditory	0.0001	0.7293
Somatomotor	0.0001	0.994	DefaultC	0.1596	0.04*	DorsalDMN	0.0159	0.6573
CingOperc	0.0359	0.8991	Language	0.2161	0.002*	HighVisual	0.1117	0.0919
DorsAttn	0.1987	0.039*	ControlA	0.0721	0.3966	Language	0.2333	0.002*
Language	0.2873	0.004*	ControlB	0.0215	0.9181	LECN	0.0028	0.8871
FrontPar	0.0556	0.9151	ControlC	0.0001	0.991	PostSal	0.0191	0.4376
Auditory	0.0001	0.7073	Sal/VenAttnA	0.0072	0.9421	Precuneus	0.0001	0.8392
Default	0.1666	0.3796	Sal/VenAttnB	0.0986	0.3726	PrimVisual	0.0001	0.5734
PostMulti	0.0896	0.042*	DorsAttnA	0.1654	0.05*	RECN	0.0043	0.8831
VentMulti	0.0565	0.2058	DorsAttnB	0.0014	0.9271	Sensorimotor	0.0001	0.8302
OrbitAffective	0.0001	0.5994	Auditory	0.0787	0.2827	VentralDMN	0.0887	0.2288
-	–	–	SomatomotorA	0.0001	0.9491	Visuospatial	0.078	0.3107
-	–	–	SomatomotorB	0.0001	0.9161	-	–	–
-	–	–	VisualA	0.255	0.0599	-	–	–
-	–	–	VisualB	0.0224	0.6094	-	–	–
-	–	–	VisualC	0.1095	0.1179	-	–	–

Yeo2011-17			Shen2013-268-8			Laird2011-20		
Name	Dice	P value	Name	Dice	P value	Name	Dice	P value
VisualA	0.2394	0.1319	MedFront	0.2558	0.025*	Emo/Interoception1	0.2264	0.2008
VisualB	0.0161	0.5974	FrontPar	0.1419	0.3806	Emo/Interoception2	0.0318	0.9421
SomatomotorA	0.0001	0.974	Default	0.138	0.2797	Emo/Interoception3	0.0408	0.6593
SomatomotorB	0.0001	0.959	Motor	0.0378	0.9241	Emo/Interoception4	0.1433	0.5085
DorsAttnA	0.2387	0.043*	VisualA	0.0899	0.3926	Mot/Visspatial1	0.0425	0.8851
DorsAttnB	0.0305	0.6474	VisualB	0.1183	0.1768	Mot/Visspatial2	0.1076	0.5524
Sal/VenAttnA	0.0178	0.8861	VisAssoc	0.2809	0.0629	Mot/Visspatial3	0.0995	0.6484
Sal/VenAttnB	0.0422	0.6204	SalSubcor	0.0482	0.8691	Mot/Visspatial4	0.1198	0.4545
LimbicA	0.0419	0.3327	-	–	–	Visual1	0.4981	0.001*
LimbicB	0.0002	0.7383	-	–	–	Visual2	0.298	0.1049
ControlC	0.0001	0.8611	-	–	–	Visual3	0.1675	0.2857
ControlA	0.1366	0.1648	-	–	–	DivergentCog1	0.2329	0.2038
ControlB	0.0129	0.9151	-	–	–	DivergentCog3	0.2038	0.3497
TempPar	0.2487	0.018*	-	–	–	DivergentCog4	0.1887	0.2458
DefaultC	0.1204	0.0649	-	–	–	DivergentCog5	0.0705	0.7323
DefaultA	0.0693	0.6074	-	–	–	DivergentCog6	0.2204	0.0699
DefaultB	0.2053	0.032*	-	–	–	-	–	–

Supplementary Table 4 (continued).

Laumann2015-12			Gordon2017-17		
Name	Dice	P value	Name	Dice	P value
Default	0.094	0.6364	Default	0.0972	0.6953
Visual	0.2123	0.2228	LatVis	0.2355	0.1638
FrontPar	0.1293	0.2677	FrontPar	0.1321	0.3087
DorsAttn	0.1371	0.2697	MedVis	0.0024	0.6743
VentAttn	0.3729	0.002*	DorsAttn	0.1873	0.1299
Salience	0.0001	0.7253	Premotor	0.0004	0.9201
CingOperc	0.0344	0.8671	Language	0.3926	0.002*
SM	0.0001	0.981	Salience	0.0039	0.7732
LateralSM	0.0001	0.7922	CingOperc	0.0329	0.8781
Auditory	0.0004	0.8332	HandSM	0.0001	0.97
MedPar	0.0001	0.7223	FaceSM	0.0001	0.7552
ParOcc	0.1264	0.014*	Auditory	0.0007	0.8591
-	–	–	AntMTL	0.0329	0.3536
-	–	–	PostMTL	0.0007	0.5594
-	–	–	ParMemory	0.0001	0.7283
-	–	–	Context	0.1473	0.029*
-	–	–	FootSM	0.0001	0.8651

Supplementary Table 5. Network spatial correspondence between UKB ICA component 5 and networks from 8 atlases (Dice coefficient and corresponding *p*-values).

Glasser2016-360+Ji2019-12

Name	Dice	P value
Visual1	0.2671	0.003*
Visual2	0.3304	0.0549
Somatomotor	0.2149	0.3966
CingOperc	0.1867	0.4745
DorsAttn	0.0375	0.8831
Language	0.0285	0.8951
FrontPar	0.0858	0.952
Auditory	0.0538	0.3357
Default	0.087	0.9101
PostMulti	0.0259	0.4535
VentMulti	0.0001	0.6873
OrbitAffective	0.0057	0.6494
-	-	-
-	-	-
-	-	-
-	-	-
-	-	-

Schaefer2018-400+Kong2019-17

Name	Dice	P value
DefaultA	0.0135	0.984
DefaultB	0.0364	0.969
DefaultC	0.1202	0.1878
Language	0.0128	0.97
ControlA	0.0374	0.9471
ControlB	0.0162	0.998
ControlC	0.0594	0.6603
Sal/VenAttnA	0.1218	0.4186
Sal/VenAttnB	0.0727	0.6943
DorsAttnA	0.0341	0.9091
DorsAttnB	0.0397	0.7712
Auditory	0.0598	0.4755
SomatomotorA	0.092	0.4835
SomatomotorB	0.1627	0.1808
VisualA	0.1709	0.1788
VisualB	0.4559	0.001*
VisualC	0.0447	0.2058

Shirer2013-90-14

Name	Dice	P value
AntSal	0.0556	0.5415
Auditory	0.012	0.6094
DorsalDMN	0.0255	0.8412
HighVisual	0.0057	0.5994
Language	0.0032	0.9131
LECN	0.0054	0.9421
PostSal	0.0116	0.7063
Precuneus	0.0325	0.4795
PrimVisual	0.0701	0.004*
RECN	0.0076	0.8951
Sensorimotor	0.087	0.2138
VentralDMN	0.0938	0.2058
Visuospatial	0.0075	0.981
-	-	-
-	-	-
-	-	-
-	-	-

Yeo2011-17

Name	Dice	P value
VisualA	0.2016	0.1029
VisualB	0.4076	0.001*
SomatomotorA	0.1494	0.3776
SomatomotorB	0.1535	0.3027
DorsAttnA	0.0671	0.6234
DorsAttnB	0.0642	0.6154
Sal/VenAttnA	0.1453	0.2927
Sal/VenAttnB	0.0882	0.5834
LimbicA	0.0001	0.8142
LimbicB	0.0001	0.7832
ControlC	0.0528	0.4176
ControlA	0.0185	0.985
ControlB	0.019	0.972
TempPar	0.003	0.8861
DefaultC	0.0913	0.1069
DefaultA	0.0274	0.9271
DefaultB	0.0456	0.9451

Shen2013-268-8

Name	Dice	P value
MedFront	0.0727	0.9331
FrontPar	0.0155	0.996
Default	0.0897	0.5395
Motor	0.2258	0.5564
VisualA	0.4734	0.001*
VisualB	0.1176	0.1109
VisAssoc	0.0645	0.7423
SalSubcor	0.2016	0.4476
-	-	-
-	-	-
-	-	-
-	-	-
-	-	-
-	-	-
-	-	-
-	-	-
-	-	-

Laird2011-20

Name	Dice	P value
Emo/Interoception1	0.0705	0.9201
Emo/Interoception2	0.0923	0.8801
Emo/Interoception3	0.0552	0.7213
Emo/Interoception4	0.1537	0.8791
Mot/Visspatial1	0.2776	0.2388
Mot/Visspatial2	0.2667	0.2767
Mot/Visspatial3	0.2032	0.5774
Mot/Visspatial4	0.1933	0.3317
Visual1	0.2736	0.2178
Visual2	0.2209	0.1139
Visual3	0.5793	0.001*
DivergentCog1	0.2408	0.4655
DivergentCog3	0.0883	0.957
DivergentCog4	0.1039	0.8242
DivergentCog5	0.1916	0.4575
DivergentCog6	0.1137	0.9301
-	-	-

Supplementary Table 5 (continued).

Laumann2015-12			Gordon2017-17		
Name	Dice	P value	Name	Dice	P value
Default	0.0602	0.9371	Default	0.0452	0.998
Visual	0.5032	0.005*	LatVis	0.3346	0.041*
FrontPar	0.0345	0.9401	FrontPar	0.0261	0.995
DorsAttn	0.0553	0.8801	MedVis	0.2568	0.002*
VentAttn	0.0144	0.962	DorsAttn	0.056	0.8192
Salience	0.0161	0.4995	Premotor	0.0245	0.7742
CingOperc	0.2082	0.2967	Language	0.0244	0.9221
SM	0.1602	0.3826	Salience	0.0624	0.4615
LateralSM	0.08	0.2118	CingOperc	0.1908	0.3477
Auditory	0.0835	0.3646	HandSM	0.0873	0.2927
MedPar	0.0322	0.2997	FaceSM	0.0775	0.1788
ParOcc	0.0725	0.2238	Auditory	0.1129	0.2957
-	–	–	AntMTL	0.0001	0.8312
-	–	–	PostMTL	0.0059	0.6833
-	–	–	ParMemory	0.0364	0.3337
-	–	–	Context	0.1215	0.0519
-	–	–	FootSM	0.0741	0.4086

Supplementary Table 6. Network spatial correspondence between UKB ICA component 3 and networks from 8 atlases (Dice coefficient and corresponding *p*-values).

Glasser2016-360+Ji2019-12			Schaefer2018-400+Kong2019-17			Shirer2013-90-14		
Name	Dice	P value	Name	Dice	P value	Name	Dice	P value
Visual1	0.0168	0.9061	DefaultA	0.0492	0.991	AntSal	0.061	0.5115
Visual2	0.107	0.7433	DefaultB	0.0938	0.8472	Auditory	0.0387	0.3337
Somatomotor	0.2585	0.3696	DefaultC	0.0532	0.8811	DorsalDMN	0.0756	0.5315
CingOperc	0.4221	0.001*	Language	0.0303	0.999	HighVisual	0.0016	0.9321
DorsAttn	0.2233	0.001*	ControlA	0.1376	0.045*	Language	0.0284	0.7552
Language	0.0383	0.993	ControlB	0.0612	0.992	LECN	0.0243	0.9121
FrontPar	0.203	0.7093	ControlC	0.0752	0.8092	PostSal	0.0844	0.001*
Auditory	0.0324	0.8581	Sal/VenAttnA	0.2207	0.001*	Precuneus	0.0388	0.6214
Default	0.1409	0.99	Sal/VenAttnB	0.2327	0.001*	PrimVisual	0.0016	0.8751
PostMulti	0.0456	0.1199	DorsAttnA	0.2225	0.001*	RECN	0.0367	0.6983
VentMulti	0.0381	0.6014	DorsAttnB	0.1886	0.001*	Sensorimotor	0.0114	0.9371
OrbitAffective	0.0291	0.4615	Auditory	0.0646	0.7692	VentralDMN	0.0948	0.2028
-	–	–	SomatomotorA	0.0818	0.7702	Visuospatial	0.1842	0.001*
-	–	–	SomatomotorB	0.0718	0.6863	-	–	–
-	–	–	VisualA	0.126	0.3307	-	–	–
-	–	–	VisualB	0.0223	0.9351	-	–	–
-	–	–	VisualC	0.0019	0.9121	-	–	–

Yeo2011-17			Shen2013-268-8			Laird2011-20		
Name	Dice	P value	Name	Dice	P value	Name	Dice	P value
VisualA	0.0145	0.973	MedFront	0.1502	0.9371	Emo/Interoception1	0.2306	0.6573
VisualB	0.011	0.97	FrontPar	0.2351	0.3417	Emo/Interoception2	0.2422	0.5065
SomatomotorA	0.1199	0.7203	Default	0.1019	0.8012	Emo/Interoception3	0.121	0.2807
SomatomotorB	0.1278	0.6693	Motor	0.4274	0.0779	Emo/Interoception4	0.3903	0.0579
DorsAttnA	0.2025	0.002*	VisualA	0.0302	0.963	Mot/Visspatial1	0.3045	0.1908
DorsAttnB	0.2177	0.001*	VisualB	0.0044	0.962	Mot/Visspatial2	0.428	0.005*
Sal/VenAttnA	0.2843	0.001*	VisAssoc	0.2464	0.022*	Mot/Visspatial3	0.4858	0.001*
Sal/VenAttnB	0.1447	0.1369	SalSubcor	0.2676	0.0539	Mot/Visspatial4	0.2043	0.5654
LimbicA	0.0504	0.7602	-	–	–	Visual1	0.3653	0.1808
LimbicB	0.0383	0.7083	-	–	–	Visual2	0.085	0.8022
ControlC	0.0683	0.3956	-	–	–	Visual3	0.0767	0.9411
ControlA	0.1944	0.005*	-	–	–	DivergentCog1	0.3851	0.5185
ControlB	0.0581	0.984	-	–	–	DivergentCog3	0.3997	0.0929
TempPar	0.0277	0.7692	-	–	–	DivergentCog4	0.2575	0.4805
DefaultC	0.024	0.7283	-	–	–	DivergentCog5	0.3691	0.0589
DefaultA	0.0732	0.971	-	–	–	DivergentCog6	0.2907	0.0649
DefaultB	0.0853	0.9201	-	–	–	-	–	–

Supplementary Table 6 (continued).

Laumann2015-12			Gordon2017-17		
Name	Dice	P value	Name	Dice	P value
Default	0.1147	0.986	Default	0.1273	0.988
Visual	0.0438	0.971	LatVis	0.035	0.9391
FrontPar	0.1773	0.1618	FrontPar	0.2354	0.0759
DorsAttn	0.312	0.001*	MedVis	0.0105	0.9401
VentAttn	0.0491	0.8811	DorsAttn	0.263	0.001*
Salience	0.0132	0.5974	Premotor	0.141	0.001*
CingOperc	0.3675	0.001*	Language	0.0586	0.8801
SM	0.1569	0.5574	Salience	0.0428	0.7952
LateralSM	0.0539	0.5734	CingOperc	0.3867	0.001*
Auditory	0.0767	0.6154	HandSM	0.0645	0.5764
MedPar	0.0331	0.4775	FaceSM	0.0368	0.6424
ParOcc	0.0551	0.3377	Auditory	0.081	0.6923
-	–	–	AntMTL	0.0369	0.7892
-	–	–	PostMTL	0.001	0.8162
-	–	–	ParMemory	0.0425	0.4356
-	–	–	Context	0.0275	0.7632
-	–	–	FootSM	0.0555	0.7403

Supplementary Figure 1. Fifteen clusters with network names. Each network was named as the atlas abbreviation followed by the network name defined by the atlas. For example, "TY17_DefaultB" is the DefaultB network from Yeo2011 17-network atlas. See Supplemental Table 1 for the atlas abbreviation. The nested stochastic block model with 15 clusters was applied onto the network similarity matrix where Dice coefficients were calculated between each pair of networks from different atlases. We note that this clustering is not meant to generate consensus networks across atlases, but simply a means to examine spatial correspondence across atlases.

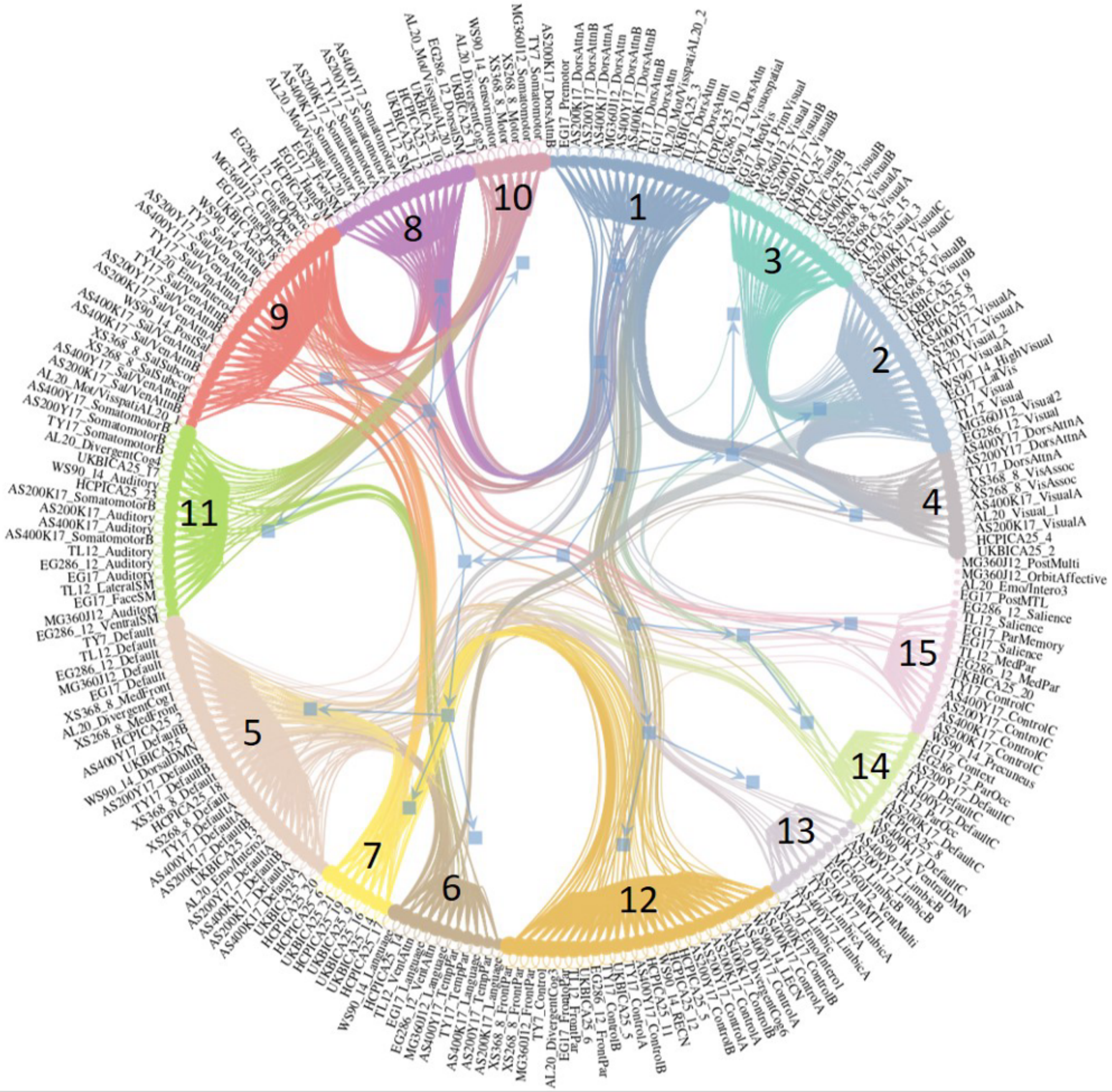

Supplementary Figure 2. The UKB ICA z-stat maps with 21 good components (https://www.fmrib.ox.ac.uk/ukbiobank/group_means/rfMRI_GoodComponents_d25_v1.txt) were thresholded by FSL melodic mixture-modeling threshold 0.6. The component 16 corresponds to cerebellum and was further excluded, resulting in 20 thresholded UKB ICA maps.

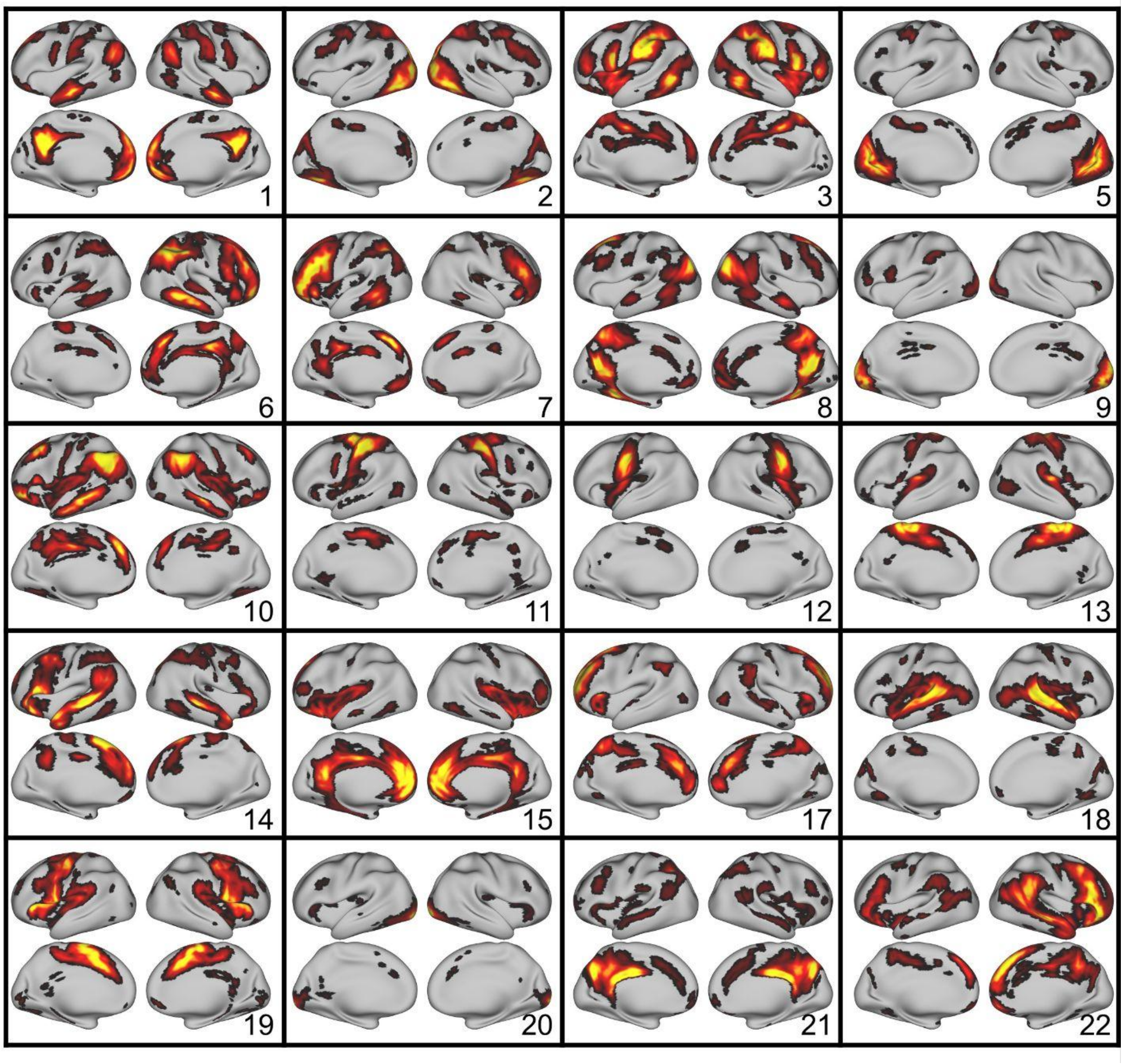

Supplementary Figure 3. The HCP ICA z-stat maps with 20 cortical components. The HCP group ICA-25 maps "melodic_IC.dscalar.nii" from HCPS1200 release were thresholded by FSL melodic mixture-modeling threshold 0.6. Five components (16, 21, 22, 24, 25) corresponding to subcortical regions were excluded, resulting in 20 thresholded HCP ICA maps.

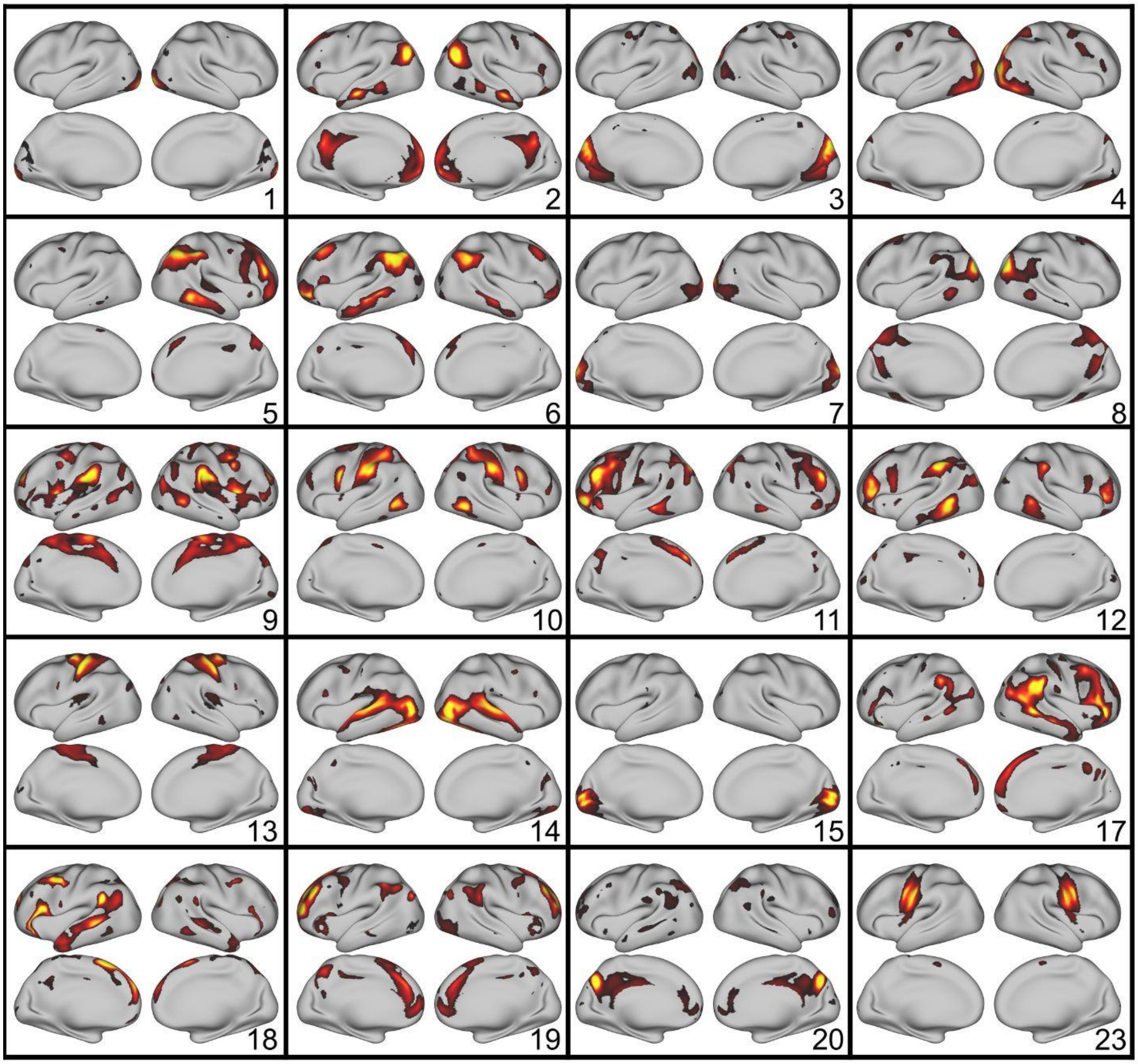

Supplement: Supplementary file 1 — Supplementary Information [file 41467_2025_58176_MOESM1_ESM.pdf]
