# Supplementary material for: A network correspondence toolbox for quantitative evaluation of novel neuroimaging results
Source: Nat Commun. 2025 Mar 25;16:2930. doi: 10.1038/s41467-025-58176-9 (PMC11937327; doi:10.1038/s41467-025-58176-9)
Supplement: Supplementary file 2 — Reporting Summary [file 41467_2025_58176_MOESM2_ESM.pdf]

nature portfolio

Corresponding author(s): Uddin, Lucina Q.

Last updated by author(s): Feb 1, 2025

# Reporting Summary

Nature Portfolio wishes to improve the reproducibility of the work that we publish. This form provides structure for consistency and transparency in reporting. For further information on Nature Portfolio policies, see our Editorial Policies and the Editorial Policy Checklist.

## Statistics

For all statistical analyses, confirm that the following items are present in the figure legend, table legend, main text, or Methods section.

| n/a | Confirmed | |
|---|---|---|
| ☐ | ☒ | The exact sample size (*n*) for each experimental group/condition, given as a discrete number and unit of measurement |
| ☒ | ☐ | A statement on whether measurements were taken from distinct samples or whether the same sample was measured repeatedly |
| ☐ | ☒ | The statistical test(s) used AND whether they are one- or two-sided<br>*Only common tests should be described solely by name; describe more complex techniques in the Methods section.* |
| ☒ | ☐ | A description of all covariates tested |
| ☐ | ☒ | A description of any assumptions or corrections, such as tests of normality and adjustment for multiple comparisons |
| ☐ | ☒ | A full description of the statistical parameters including central tendency (e.g. means) or other basic estimates (e.g. regression coefficient) AND variation (e.g. standard deviation) or associated estimates of uncertainty (e.g. confidence intervals) |
| ☐ | ☒ | For null hypothesis testing, the test statistic (e.g. *F*, *t*, *r*) with confidence intervals, effect sizes, degrees of freedom and *P* value noted<br>*Give P values as exact values whenever suitable.* |
| ☒ | ☐ | For Bayesian analysis, information on the choice of priors and Markov chain Monte Carlo settings |
| ☒ | ☐ | For hierarchical and complex designs, identification of the appropriate level for tests and full reporting of outcomes |
| ☒ | ☐ | Estimates of effect sizes (e.g. Cohen's *d*, Pearson's *r*), indicating how they were calculated |

*Our web collection on statistics for biologists contains articles on many of the points above.*

## Software and code

Policy information about availability of computer code

| | |
|---|---|
| Data collection | Data collection software includes the Connectome Workbench, ANTs, FSL, and FreeSurfer. |
| Data analysis | Atlas projection scripts rely on Connectome Workbench, ANTs and FSL. Data analysis and visualization scripts rely on open-source Python packages. |

For manuscripts utilizing custom algorithms or software that are central to the research but not yet described in published literature, software must be made available to editors and reviewers. We strongly encourage code deposition in a community repository (e.g. GitHub). See the Nature Portfolio guidelines for submitting code & software for further information.

## Data

Policy information about availability of data

All manuscripts must include a data availability statement. This statement should provide the following information, where applicable:

- Accession codes, unique identifiers, or web links for publicly available datasets
- A description of any restrictions on data availability
- For clinical datasets or third party data, please ensure that the statement adheres to our policy

*Provide your data availability statement here.*



April 2023

## Research involving human participants, their data, or biological material

Policy information about studies with human participants or human data. See also policy information about sex, gender (identity/presentation), and sexual orientation and race, ethnicity and racism.

| | |
|---|---|
| Reporting on sex and gender | Not applicable, we did not recruit human participants in this analysis. |
| Reporting on race, ethnicity, or other socially relevant groupings | Not applicable, we did not recruit human participants in this analysis. |
| Population characteristics | Not applicable, we did not recruit human participants in this analysis. |
| Recruitment | Not applicable, we did not recruit human participants in this analysis. |
| Ethics oversight | Not applicable, we did not recruit human participants in this analysis. |

Note that full information on the approval of the study protocol must also be provided in the manuscript.

# Field-specific reporting

Please select the one below that is the best fit for your research. If you are not sure, read the appropriate sections before making your selection.

☒ Life sciences ☐ Behavioural & social sciences ☐ Ecological, evolutionary & environmental sciences

For a reference copy of the document with all sections, see nature.com/documents/nr-reporting-summary-flat.pdf

# Life sciences study design

All studies must disclose on these points even when the disclosure is negative.

| | |
|---|---|
| Sample size | We included 16 atlases with 230 different networks in this analysis. HCP task contrast maps are averaged across 997 subjects. |
| Data exclusions | We excluded noisy components for ICA maps. |
| Replication | The present study proposes a toolbox and does not include any experimental analyses. The results presented in this manuscript can be replicated by the released online toolbox through Github (https://github.com/rubykong/cbig_network_correspondence) or the Python Package Index (PyPI; https://pypi.org/project/cbig-network-correspondence). |
| Randomization | The present study proposes a toolbox and does not include any experimental analyses. We did not include any participants in this study, therefore, there was no randomization in terms of participants. The atlases we included in this paper are from different published papers derived by different datasets. These atlases are also in different spaces. |
| Blinding | Not applicable. The present study proposes a toolbox and does not include any participants. |

# Reporting for specific materials, systems and methods

We require information from authors about some types of materials, experimental systems and methods used in many studies. Here, indicate whether each material, system or method listed is relevant to your study. If you are not sure if a list item applies to your research, read the appropriate section before selecting a response.

### Materials & experimental systems

| n/a | Involved in the study | |
|---|---|---|
| ☒ | ☐ | Antibodies |
| ☒ | ☐ | Eukaryotic cell lines |
| ☒ | ☐ | Palaeontology and archaeology |
| ☒ | ☐ | Animals and other organisms |
| ☒ | ☐ | Clinical data |
| ☒ | ☐ | Dual use research of concern |
| ☒ | ☐ | Plants |

### Methods

| n/a | Involved in the study | |
|---|---|---|
| ☒ | ☐ | ChIP-seq |
| ☒ | ☐ | Flow cytometry |
| ☐ | ☒ | MRI-based neuroimaging |

## Plants

| | |
|---|---|
| Seed stocks | NA |
| Novel plant genotypes | NA |
| Authentication | NA |

## Magnetic resonance imaging

### Experimental design

| | |
|---|---|
| Design type | Functional MRI. The atlases included in the present study are generated by functional MRI data. |
| Design specifications | Not applicable. The present study proposes a toolbox and does not include any experimental analyses. |
| Behavioral performance measures | Not applicable. The present study proposes a toolbox and does not include any experimental analyses. |

### Acquisition

| | |
|---|---|
| Imaging type(s) | Not applicable. The present study proposes a toolbox and does not include any images. |
| Field strength | Not applicable. The present study proposes a toolbox and does not include any images. |
| Sequence & imaging parameters | Not applicable. The present study proposes a toolbox and does not include any images. |
| Area of acquisition | Not applicable. The present study proposes a toolbox and does not include any images. |

Diffusion MRI ☐ Used ☒ Not used

### Preprocessing

| | |
|---|---|
| Preprocessing software | Not applicable. The present study proposes a toolbox and does not include any images. Therefore, there is no data preprocessing in the manuscript. However, we use FSL, ANTs, and Connectome Workbench for atlas projection. |
| Normalization | Not applicable. The present study proposes a toolbox and does not include any images. Therefore, there is no data normalization. |
| Normalization template | Not applicable. The present study proposes a toolbox and does not include any images. Therefore, there is no data normalization. |
| Noise and artifact removal | Not applicable. The present study proposes a toolbox and does not include any images. Therefore, there is no noise and artifact removal. |
| Volume censoring | Not applicable. The present study proposes a toolbox and does not include any images. Therefore, there is no volume censoring. |

### Statistical modeling & inference

| | |
|---|---|
| Model type and settings | *Specify type (mass univariate, multivariate, RSA, predictive, etc.) and describe essential details of the model at the first and second levels (e.g. fixed, random or mixed effects; drift or auto-correlation).* |
| Effect(s) tested | *Define precise effect in terms of the task or stimulus conditions instead of psychological concepts and indicate whether ANOVA or factorial designs were used.* |

Specify type of analysis: ☒ Whole brain ☐ ROI-based ☐ Both

| | |
|---|---|
| Statistic type for inference (See Eklund et al. 2016) | We perform spin test for statistical test |
| Correction | We provide a toolbox showing the p-value for Dice overlap. We didn't perform correction. |

## Models & analysis

| n/a | Involved in the study |
|---|---|
| ☒ | ☐ Functional and/or effective connectivity |
| ☒ | ☐ Graph analysis |
| ☒ | ☐ Multivariate modeling or predictive analysis |
